# Identification of a Person in a Trajectory Based on Wearable Sensor Data Analysis

**DOI:** 10.3390/s24113680

**Published:** 2024-06-06

**Authors:** Jinzhe Yan, Masahiro Toyoura, Xiangyang Wu

**Affiliations:** 1 Department of Computer Science, Hangzhou Dianzi University, Hangzhou 310018, China; 2 Department of Computer Science and Engineering, University of Yamanashi, 4-3-11 Takeda, Kofu 400-8511, Japan

**Keywords:** deep learning, edge computing, human trajectory, IMU sensor, multimodal sensor data analysis, time series, transformer

## Abstract

Human trajectories can be tracked by the internal processing of a camera as an edge device. This work aims to match peoples’ trajectories obtained from cameras to sensor data such as acceleration and angular velocity, obtained from wearable devices. Since human trajectory and sensor data differ in modality, the matching method is not straightforward. Furthermore, complete trajectory information is unavailable; it is difficult to determine which fragments belong to whom. To solve this problem, we newly proposed the *SyncScore model* to find the similarity between a unit period trajectory and the corresponding sensor data. We also propose a *Likelihood Fusion algorithm* that systematically updates the similarity data and integrates it over time while keeping other trajectories in mind. We confirmed that the proposed method can match human trajectories and sensor data with an accuracy, a sensitivity, and an F1 of 0.725. Our models achieved decent results on the UEA dataset.

## 1. Introduction

The prevalence of surveillance cameras makes it possible to track individuals in various settings such as fitness gyms, classrooms, and nursing facilities. This data not only serves security purposes but also provides valuable insights for individuals wishing to review their own actions. It is possible to infer from the trajectory how many minutes a user spends on a particular fitness center machine, how long a certain group spends in a classroom discussion, or how long a user interacts with others in a care facility lounge. By matching an individual’s trajectories with other collected data, such as health data, trajectories can be used to determine strategies such as feedback to maximize the effectiveness of training, learning, and care interventions.

Data from wearable sensors alone can provide information on exercise duration and intensity, stationary time periods, GPS location, etc., and can be used for the purposes described above. Sleep duration and walking distance are also being measured by commercial wearable watches. This information contains important insights for maintaining good health. One thing that cannot be obtained from wearable sensor data alone is information on the trajectory of movement in a room or building. This information may be obtained from cameras installed in the room or the building, and can be used in a complementary manner to the wearable sensor data.

**Figure 1:**
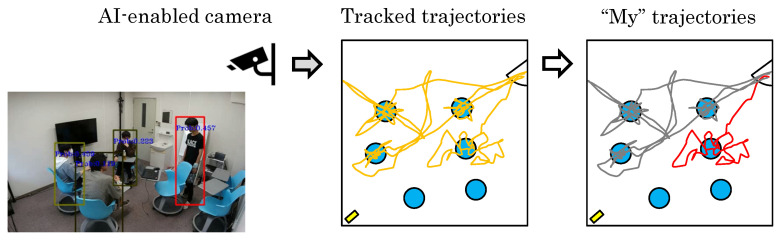
A situation where a specific person wants to distinguish his/her trajectories from all other trajectories. An AI camera provides a mixture of all trajectories. By extracting the person’s trajectory from these trajectories, it is possible to analyze the person’s behavior. In particular, the person’s own trajectory provides a record of which room the person was moving in and how he/she was moving.

Figure 1 shows a hypothetical situation. Here, obtaining all the trajectories in the scene does not sufficiently reflect an individual’s actions. While all trajectories are available, each individual needs to know theirs. The trajectories for all individuals are obtained in a mixed state, and some are lost or misextracted. By identifying a specific person’s trajectory from others, it becomes possible to understand that person’s behavior. Furthermore, if the trajectories can be broken down individually, it will be possible to understand how people behave as a group. In this work, we focus on extracting the trajectory of the user we wish to analyze.

A naive idea is to track the faces in the video, but there are several problems with this. First of all, if a person turns one’s back to the camera, we cannot see that person’s face. In addition, if data management is based on privacy by design, a policy may be adopted to preserve only the trajectory and not the images themselves.

Another idea is to track people by their clothes, but this will be difficult when there is no diversity in the clothing of the people in a fitness gym or a care facility. In addition, no images will be saved because of privacy by design.

**Figure 2:**
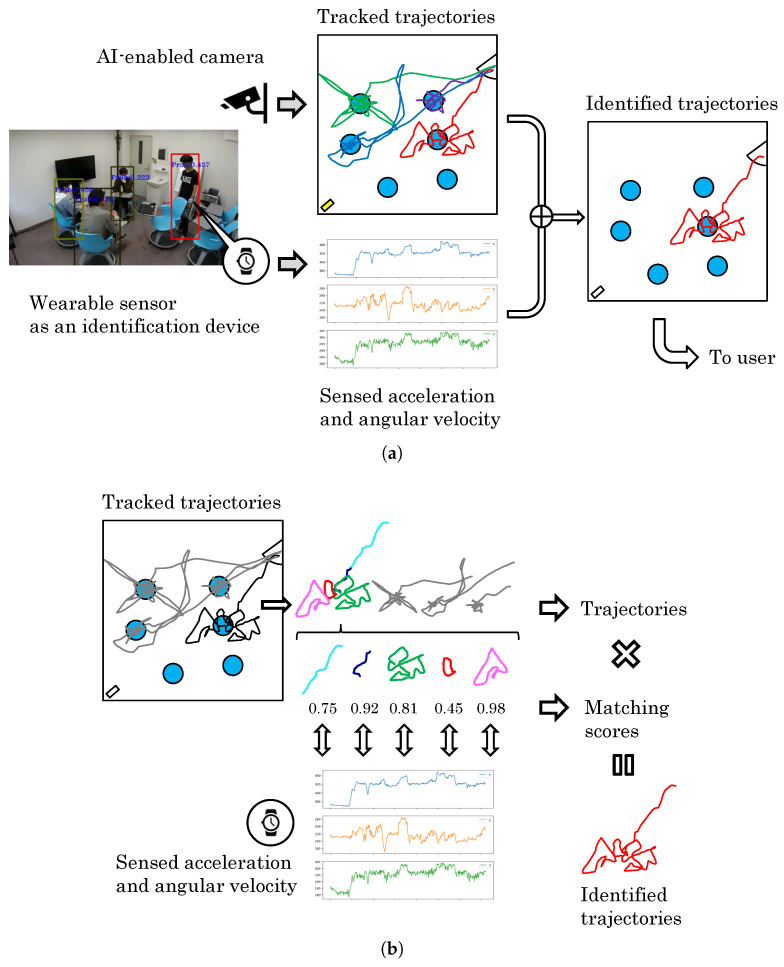
Overview of service and matching process. By matching the trajectories with the sensor signals of the wearable device, only the trajectory of the person who owns the wearable device is extracted. The owner of the device can extract his or her own trajectories from a large number of trajectories. The trajectories are observed as a mixture of the segmented trajectories. The proposed method computes a match score between the sensor signal and all corresponding segment trajectories, and aims to retrieve all corresponding trajectories. (**a**) Overview of the services envisioned. (**b**) Overview of the matching process.

We propose a method of directly matching trajectory data with inertial data from a wearable watch. This method does not require any video saving as shown in Figure 2. From a privacy-by-design point of view, it is desirable to convert the trajectory data on the edge device. A new problem is that inertial data from the wearable watch may be added as a new condition, but this condition may not be a problem in many cases where the user wants to acquire his/her own trajectories while standing in front of a surveillance camera, or use his/her own trajectories for some kind of analysis.

However, to the best of the authors’ knowledge, no matching algorithm can solve such a problem. This work provides a new perspective and method for matching trajectories and sensor data, and provides a valuable basis for further research and applications in this area as well as a tool to support personalized services by enabling the analysis of individual behavior and the design of intelligent systems.

Although there are many related methods of image feature-based person re-identification [1,2,3], these methods aim to find the same person in different images. Person re-identification is achieved by extracting visual features such as clothing and facial features in one frame image and searching for a person with the same features in another frame image. In this work, instead of extracting features from individual frames, we aim to achieve personal recognition by calculating the likelihood of similarity between a person’s trajectory and simultaneously obtained sensor data. Deep neural networks can be used to calculate the similarity likelihood.

While actual attempts to match trajectories to sensor data provide uninterrupted sensor data, it is often the case that a person is not tracked continuously by the camera and is re-tracked after a few frames. As a result, a person’s trajectory will be fragmented. It is necessary to match sensor data even from such fragmented trajectories. We propose a method for matching both trajectory and sensor data at short intervals, and integrating the results into a whole sequence. We also propose a method for updating the matching likelihood by looking at the whole set of trajectories, based on the fact that, when multiple trajectories are observed, there will be at most one trajectory that corresponds to each of them.

In our proposed method, the matching likelihood is obtained from trajectory data and wearable sensor data. Both short-time trajectory data and sensor data are input into a deep neural network to obtain the matching likelihood. Next, the short-time trajectories are integrated to obtain the likelihood of the sensor data for a series of trajectories tracking the same person. Finally, the trajectory with the highest likelihood is selected to complete the matching of trajectory and sensor data.

The main challenge lies in the mismatch between the signals obtained from the wearable watch and the individual trajectories, which belong to different modalities. Additionally, occlusions can cause fragmentation in the individual trajectories, necessitating a method of collecting and integrating these fragmented trajectories using the information from the watch signal.

The contributions of this paper are as follows:

1. We constructed a deep learning network-based *SyncScore model* (Section 3.2) to recognize the degree of matching between trajectory data and sensor data at each unit time period. A newly proposed Fusion Feature module (Section 3.2.1) and SecAttention module (Section 3.2.2) contribute to improve the matching accuracy.
2. We developed a *Likelihood Fusion algorithm* (Section 3.3) that integrates the degree of matching between the trajectory data and sensor data for the entire trajectory by incrementally updating it while referring to the status of other trajectories. Update Rules (Section 3.3.1 and Section 3.3.2) contribute to integrating short-period likelihood into full-period likelihood.
3. We created a dataset specifically designed for our research purposes (Section 4.1). With this dataset, we were able to evaluate the performance of our proposed method in specific scenarios and achieved satisfactory results.
4. We experimented with some of the proposed modules on the UEA dataset and achieved excellent results (Section 4.2).

Section 2 lists related work and identifies remaining issues, and Section 3 describes the methodology. Section 4 describes database construction and experiments, and Section 5 summarizes the conclusions.

## 2. Related Work

Expansion on previous work and addressing limitations: Previous research has primarily focused on unimodal data, relying solely on image or video data. While significant progress has been made in related tasks, there is a lack of specific methods addressing the proposed task. Moreover, there is limited in-depth research on effectively utilizing and processing multi-channel data. The challenges associated with multi-channel tasks include the complexity of the data, the high dimensionality of the feature space, and the heterogeneity of the modalities.

To overcome these challenges, several aspects need to be considered:

- Data heterogeneity: Different modalities can produce diverse types of data, such as images, audio, text, or sensor readings. Developing a common representation and effectively integrating these heterogeneous modalities is a complex task.
- Feature extraction: Multi-channel tasks often require extracting features from multiple modalities, which can be time-consuming and computationally expensive. Selecting informative features that capture the essence of each modality is crucial for achieving accurate results.
- Alignment and synchronization: Modalities may not be perfectly aligned in time, leading to misalignment and reduced prediction accuracy. Synchronizing the modalities, especially in large datasets, presents a significant challenge.

By addressing these limitations and considering the complexities associated with multi-channel data, our proposed algorithm aims to provide an effective solution for selecting and obtaining individual trajectories with high precision while ensuring privacy. The algorithm utilizes the information from the wearable watch signals and integrates fragmented trajectories, ultimately enhancing the usability and privacy preservation of the data.

### 2.1. Person Identification by Appearance

Research work in the field of person identification, including deep learning-based face recognition, face embedding, face detection and alignment, person re-identification, and so forth, has proposed various innovative models and methods. Taigman et al. [4] proposed the DeepFace model, which achieved near-human-level facial verification performance through deep learning techniques. Their proposed model is trained on a large-scale facial image dataset to achieve efficient face verification by learning a compact representation of facial features.

Sun et al. [5] proposed a deep learning-based facial representation method to learn facial feature representations by predicting large-scale face categories. Their method utilizes deep neural networks to embed facial images into a high-dimensional feature space for face recognition and related tasks. We were inspired by this and concluded that we could also map our data into a high-dimensional feature space for further processing. Xiao et al. [6] proposed an end-to-end deep learning method for the task of person search. Their method combines person re-identification and object detection in a unified framework for accurate person search through federated learning. Wang et al. [7] proposed a pyramid space–time aggregation method for extracting feature representations of people in videos. This method divides the video into pyramid levels of multiple spatial and temporal scales, and obtains multi-scale feature representations by extracting features at each level and aggregating them. This pyramid structure can capture character feature information on different spatial and temporal scales, thereby improving the accuracy of character re-identification. Zhou et al. [3] proposed a full-scale feature learning method for person re-identification. Their method achieves more comprehensive person re-identification by learning global and local features. We were inspired to design a fusion of global features.

### 2.2. Multidimensional Time-Series Signal Processing

Our task involves processing multidimensional time series and designing models for multiple inputs. Our research in related fields shows that dealing with multidimensional time series is an important topic.

To solve this problem, we needed to consider some key factors. First, the processing of multidimensional time series needs to take into account the structure and characteristics of the data. Time-series data often has multiple dimensions, each of which may represent a different feature or variable. Therefore, we needed to choose appropriate methods to capture the association and temporal dependencies between different dimensions. Second, for models with multiple inputs, we needed to design appropriate architectures to handle different types of inputs. This involved fusing information from multiple input sources. Feature selection and dimensionality reduction were also important steps in dealing with these multidimensional time series.

Peng et al. [8] proposed a method based on wavelet transform and neural networks to forecast multidimensional time series. This method explores the application of wavelet transform in time-series feature extraction and combines it with neural network models. Hyndman et al. [9] introduced the method of using functional data analysis (FDA) to process multidimensional time-series data. This explores how multidimensional time series can be viewed as functional data and be modeled and analyzed using FDA techniques. Wang et al. [10] explored a method for multi-step time-series forecasting based on long short-term memory networks (LSTMs). Multi-step time-series forecasting refers to predicting the value of multiple time steps in the future through historical time-series data. LSTMs are a variant of recurrent neural networks (RNNs) and are particularly suited to processing sequential data. Wang et al. [11] gave an overview of the application of deep learning to time-series classification. This overview covers processing and feature extraction methods for multidimensional time-series, and summarizes the performance and challenges of deep learning models in time-series classification tasks. We were inspired by this and decided to design a deep learning model for our task. Pham et al. [12] proposed the temporally weighted spatiotemporal explainable model (TSEM), a neural network model for processing multivariate time-series data. This model combines temporal weighting and space–time interpretability mechanisms, aiming to provide interpretability of time-series forecasts to enhance model understandability and user trust.

Numerous studies are actively being conducted to estimate the user’s behavior and health status from IMU [13], GPS [14], and biosensors [15] attached to wearable watches and other devices. There is also work on platforms to utilize this information for the remote monitoring of patients [16]. Machine learning, including deep learning, is also used in these studies. While these studies provide rough information in terms of hours and minutes to promote health, we aim to estimate activity at a more detailed level, with the limitation that it is within a specific room that can be observed by an AI camera.

In recent years, Transformer models have shown remarkable results in various fields, which has been widely recognized.

Vaswani et al. [17] proposed the Transformer model, which introduced the self-attention mechanism into sequence modeling and replaced the traditional RNN structure. This model has achieved excellent performance on tasks such as machine translation. Huang et al. [18] proposed a Transformer-based time-series modeling method, which uses an autoregressive generative process to model time-series data. The method utilizes the Transformer model to model and predict temporal dependencies in sequences. Wu et al. [19] proposed a new method using the deep Transformer model for the important time-series forecasting task of influenza epidemic trend prediction. Traditional time-series forecasting methods may have difficulty capturing the long-term dependencies and nonlinear characteristics in time series, while the deep Transformer model has strong modeling ability and adaptability. Fawaz et al. [20] realized that time-series data have unique characteristics that require specialized methods for effective analysis. They proposed the InceptionTime model, which is based on the Inception architecture and aims to address the challenges of time-series classification. They applied AlexNet, a successful image classification architecture, to time-series classification tasks.

## 3. Methods

### 3.1. Sequences of Human Trajectory and Wearable Sensor Data

Suppose that a camera capable of AI processing provides the positions of several people in an image at time *t*. Since AI processing can extract a human region as a rectangle and can estimate the depth of the region, we define a human position as a 3D position that represents the center of the rectangle.

If the positions change little in adjacent frames, then a series of human trajectories can easily be connected. At time t−Δt, if the Euclidean distance is the closest to the human position at time *t* and the distance is smaller than a certain threshold, then these positions are considered to be those of the same person. Since a human position may not be available due to occlusion or frameout, the trajectory is considered to cease and a new trajectory is considered to start when the closest distance is no longer available. A human trajectory may be confused with that of another person but, since this does not happen frequently, it is ignored in this paper. There are previous studies that determine the confusion of trajectories based on the characteristics of a person’s region [21,22].

**Figure 3:**
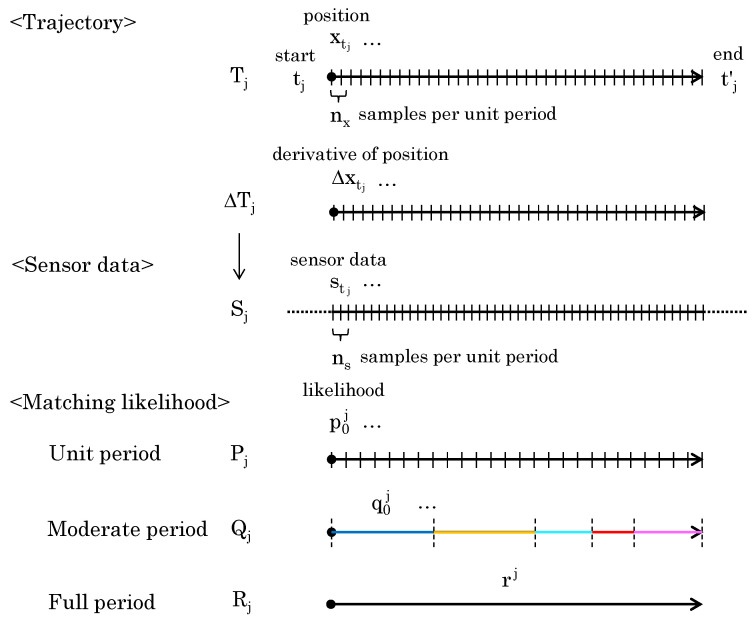
Sampling data and different period likelihood.

Figure 3 shows the definitions for the trajectory, sensor data, and the matching likelihood obtained from them. The human trajectory $T_{j}$ is defined as $T_{j}:\left\{t_{j},t_{j}^{\prime },X_{j}\right\}$, $X_{j}=\left(x_{t_{j}},\cdots ,x_{t_{j}^{\prime }}\right)$, where $t_{j}$ and $t_{j}^{\prime }$ represent the first and last time of the trajectory, $X_{j}$ is the set of 3D positions, and $x_{t_{j}}$ is the 3D positions in the first frame. The 3D position is constituted by an object’s x and y coordinates within the image plane and its depth z indicating the distance from the viewpoint. $n_{x}$ represents the number of samples per unit period; here, “per unit period” is defined as one-second trajectory length and sensor data length, which is the input for the deep neural network.

Since the absolute 3D position is not represented in the sensor data, the time derivative of the position $\Delta T_{j}$ is used instead of the 3D position. $\Delta T_{j}$ corresponds to the velocity at each time. If $\Delta x_{t_{j}}$ is the derivative of position at time $t_{j}$, then the derivative of the human trajectory $\Delta T_{j}$ is $\Delta T_{j}:\left\{t_{j},t_{j}^{\prime },\Delta X_{j}\right\}$, $\Delta X_{j}=\left(\Delta x_{t_{j}},\cdots ,\Delta x_{t_{j}^{\prime }}\right)$.

The wearable sensor data, on the other hand, is 9D data consisting of 3D acceleration, 3D angular velocity, 3D angular acceleration, and 3D magnetic field. For the acceleration and the angular velocity, the data are obtained in the sensor system. We are primarily interested in the rate of change of an object’s velocity and its rate of rotation, rather than the individual components of these vectors. Hence, through $a=\sqrt{a_{x}^{2}+a_{y}^{2}+a_{z}^{2}}$ and $\omega =\sqrt{\omega_{x}^{2}+\omega_{y}^{2}+\omega_{z}^{2}}$, we convert acceleration and angular velocity into 1D data.

As a result, the wearable sensor data are treated as 5D data at each point in time, consisting of 3D angles, processed 1D acceleration, and 1D angular velocity. It can be assumed that data from wearable sensors, unlike trajectory data, are constantly obtained without the discontinuities that happen during video capture. The wearable sensor data that corresponds to $T_{j}$ is denoted as $S_{j}:\left\{t_{j},t_{j}^{\prime },A_{j}\right\}$, $A_{j}=\left(a_{t_{j}},\cdots ,a_{t_{j}^{\prime }}\right)$, where $A_{j}$ represents the set of 5D wearable data, which serves as the input to our SyncScore. $a_{t_{j}}$ and $a_{t_{j}^{\prime }}$ are the 5D data at the first and last points in time. $n_{s}$ and $n_{x}$ represent the sampling frequency of the sensor and trajectory, respectively.$n_{s}$ is generally more than the number of frames in the trajectory ($n_{x}$) because the sampling frequency of the sensor data is generally higher than that of the camera-based human trajectory data.

For $\Delta T_{j}$ and $S_{j}$, we can estimate the matching likelihood of the trajectory and data at each unit time. This is defined as $P_{j}=\left(p_{0}^{j},\cdots \right)$. The likelihood is obtained by the deep neural networks (DNNs)—SyncScore described in Section 3.2. Finally, the likelihood obtained for the entire $\Delta T_{j}$ and $S_{j}$ is $R_{j}=\left(r^{j}\right)$. Furthermore, as a likelihood with a granularity between $P_{j}$ and $R_{j}$, the matching likelihood for a certain time period is expressed as $Q_{j}=\left(q_{0}^{j},\cdots \right)$. The method for time period division is explained in Section 3.3.

### 3.2. SyncScore Network for Unit Period Recognition

Assuming that data with the same timestamp can be extracted from $\Delta T_{j}$ and $S_{j}$, we want to examine whether they are from the same person. There are several issues that need to be addressed:

1. The start and end times are different in the trajectory and sensor data.
2. The number of frames per unit time period is different in trajectory and sensor data due to different sampling rates.
3. The lengths of the trajectories are different, so the dimension for network input is indeterminate.
4. Even during periods when sensor data is available, trajectories will be partially unavailable, such as when the person is occluded from the camera.

To address these issues, we propose a deep learning network that extracts unit period data from trajectory and sensor data for comparison. If we can determine, per unit period, whether both datasets come from the same person, we will be able to investigate data across all time periods. Since we can obtain one sample per unit period, we can prepare the number of samples needed for deep learning.

**Figure 4:**
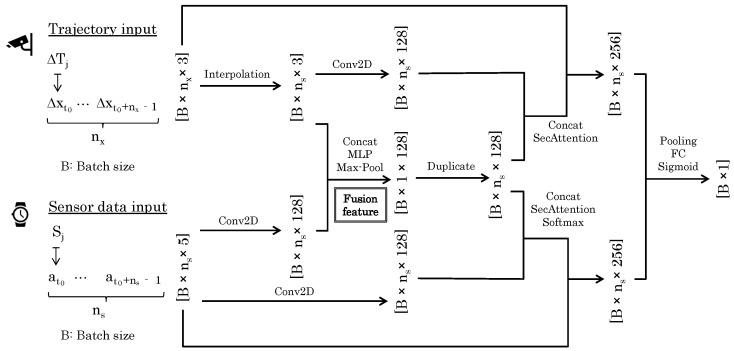
Structure of the SyncScore network.

We defined the number of the samples per unit period as $n_{c}$ for the trajectory and $n_{s}$ for the sensor data. The input dimensions were then $n_{x}\times 3$ and $n_{s}\times 5$, respectively. The output was a 1D likelihood that would tend to be one if both data originated from the same person, and zero otherwise. Within the batch size designated as *B*, we trained the network shown in Figure 4, which we named the *SyncScore network*.

The SyncScore network takes unit period trajectory $\Delta T_{j}$ and the corresponding sensor data $S_{j}$ as inputs and outputs the likelihood that they match. The training data can be obtained by having the target person wear the IMU sensor while recording the video simultaneously. Because we timestamped each piece of data during recording, we can align trajectory and sensor data through their corresponding timestamps. An annotator can easily tag the person wearing the sensor in the video by viewing footage. By training a network that outputs one when the sensor data and the trajectory of the target person are input and zero when the sensor data and the trajectory of another person are input, we will be able to determine the likelihood that the sensor data and the trajectory are from the same person when unknown data are input.

The SyncScore network is unique in several ways. First, the trajectory $\Delta T_{j}$ has a length $n_{x}$, while the sensor data $S_{j}$ has a length $n_{s}$; we interpolate along the time dimension to match the length $n_{s}$. This is achieved by interpolating additional data points between the existing points along each row. Specifically, the interpolated value *v* at a position τ in a row can be calculated using the following formula:

(1) $$v_{\tau }=v_{\tau_{1}}+\left(\tau -\tau_{1}\right)\cdot \frac{v_{\tau_{2}}-v_{\tau_{1}}}{\tau_{2}-\tau_{1}}$$

where $\tau_{1}$ and $\tau_{2}$ are the positions of the nearest known data points surrounding the interpolation point τ, and $v_{\tau_{1}}$ and $v_{\tau_{2}}$ are the values at these points. Next, a one-dimensional convolution process is performed on each of the trajectory and sensor data, and the concatenation of the two, or *Fusion Feature*, is synthesized. By aligning the two data sources, we created an integrated representation that captures the joint characteristics and facilitates a comprehensive analysis. This fusion approach enables us to consider the holistic aspects of the data and extract meaningful insights regarding their interplay.

It is necessary to match trajectory and sensor data at multiple time scales. Drawing inspiration from the InceptionTime model [20], we concatenated time-series data of different scale sizes and processed them as a single set of data. By doing so, we expected that matching results would not be compromised by short-term noise and that short-time features, which would be missed when considering only the long-term data, would also be considered.

#### 3.2.1. Fusion Feature

**Figure 5:**
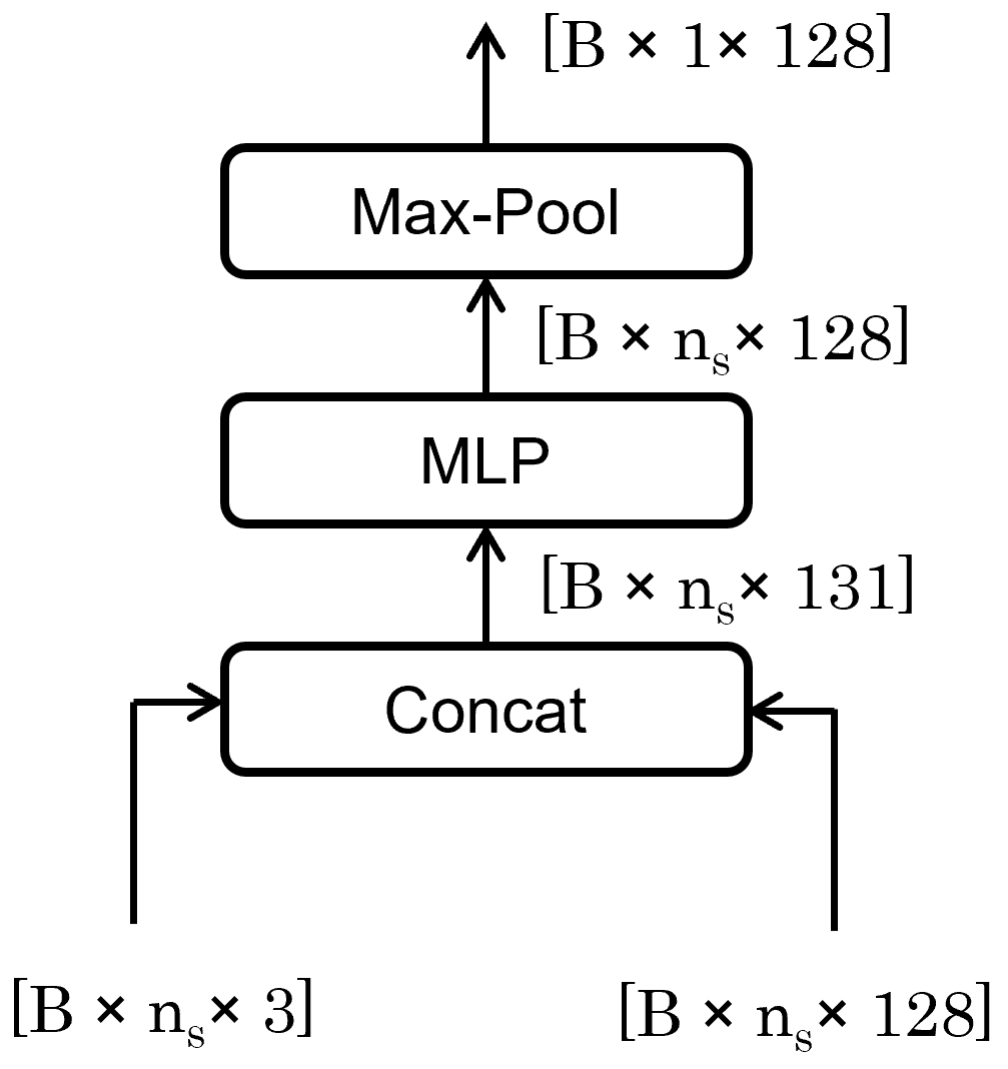
Fusion Feature architecture.

The Fusion Feature module, as shown in Figure 5, plays a pivotal role in the neural network by merging trajectory and sensor data to form a comprehensive feature set. This process involves concatenating features from both sources, enhancing the system’s state representation. Subsequent processing through a multilayer perceptron (MLP) and a max-pooling operation further refines these features by reducing dimensionality and emphasizing the most critical attributes. This streamlined approach not only reduces computational load but also mitigates the risk of overfitting, ensuring the module effectively captures essential global features for the task at hand, contingent on the integrity and synergy of the input data.

#### 3.2.2. SecAttention

In this work, we used a modified Transformer to achieve high recognition accuracy for the target data. The Transformer [17], which has a self-attention mechanism, was successful at recognitioning series data. The self-attention mechanism can adaptively calculate the attention weight of each position according to the importance ascribed to different positions in the input data. By modeling and learning the relationships between locations, we can more accurately understand and capture key dependencies in the data.

**Figure 6:**
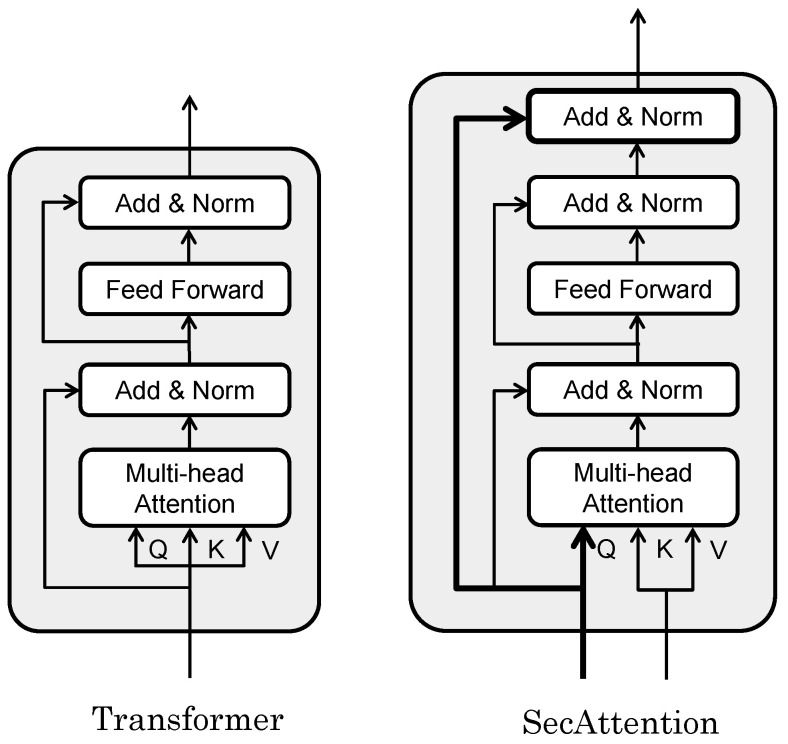
SecAttention model extended from the Transformer model.

Figure 6 shows the difference between the Transformer and our extended SecAttention. In the Transformer, the input to the block is normalized twice before output but, in our extended SecAttention, the input to the block is re-concatenated before normalization so that the input features leave the block in their original form. The input and output matrixes of the SecAttention module have the same dimensions but, by directly modeling the relationship between any two positions in the sequence, we can better capture the long-range dependencies in the sequence. The effect of this seemingly trivial improvement on the data is demonstrated numerically in the ablation study in the experimental section. Additionally, Transformer models typically employ the same input for linear transformations to derive queries, keys, and values. In contrast, our SecAttention model utilizes data transformed from features for its keys and values. For queries, we retained the original information and applied a linear transformation to it.

#### 3.2.3. Loss Function

Binary Cross-Entropy (BCE) loss is a common loss function used in binary classification tasks. In the field of machine learning, especially when dealing with binary classification, BCE is used to measure the discrepancy between the model’s predicted probabilities and the actual labels. Its primary purpose is to minimize the entropy difference between predictions and actual values, thereby enhancing the accuracy of the model’s classification. The BCE loss can be calculated using the following formula:

(2) $$\mathrm{BCE}=-\frac{1}{N}\sum_{i=1}^{N}\left[y_{i}\cdot \log\left({\hat{y}}_{i}\right)+\left(1-y_{i}\right)\cdot \log\left(1-{\hat{y}}_{i}\right)\right]$$

where *N* is the number of samples. $y_{i}$ is the actual label of the *i*-th sample, which is either 0 or 1. ${\hat{y}}_{i}$ is the model’s predicted probability of the *i*-th sample being of the positive class.

### 3.3. Likelihood Fusion Algorithm

The Likelihood Fusion algorithm is shown below. In the algorithm, the matching likelihoods obtained for each unit period are integrated step by step, and the desired likelihoods for the full period are obtained while referring to the status of other trajectories.

**Figure 7:**
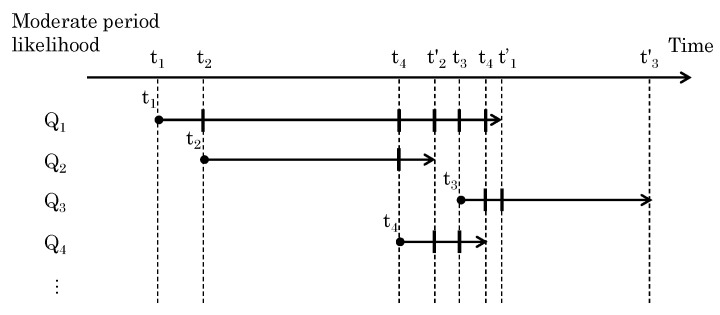
Definition of moderate period by start and end times of all trajectories.

The $Q_{j}$ in Figure 3 represents the matching likelihoods for the segmented periods in trajectory $T_{j}$. The period is defined as a single time segment when multiple trajectories have a common time period, as shown in Figure 7. When trajectory $T_{j}$ starts at time $t_{j}$ and ends at time $t_{j}^{\prime }$, then the start and end times of any given trajectory can be used to identify the time period in which multiple trajectories occur. For example, we can say that different pairs of segments share the same time period before and after the start of a given trajectory.

In each pair, a maximum of one segment corresponds to the sensor data. Additionally, trajectory $T_{j}$ partially does not correspond with the sensor data. Considering this, we updated the elements of $Q_{j}$ before integrating $Q_{j}$ into the likelihood $R_{j}$.

**Figure 8:**
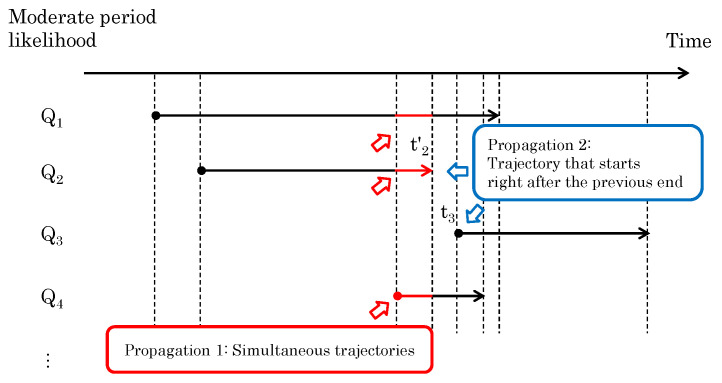
The overview of Update Rules 1 and 2 for segmented likelihoods of trajectories.

When multiple segments exist in the same time period, the one with the highest likelihood should be highlighted (Update Rule 1). When trajectories are segmented by a person’s occlusion, the segmented trajectories are treated differently. Therefore, if another trajectory with a high likelihood ends just before the start of one trajectory, the likelihood of the trajectory that begins immediately after it should be increased (Update Rule 2). The overview is shown in Figure 8, and the detailed methods are described below.

#### 3.3.1. Update Rule 1—Simultaneous Trajectories

For segmented trajectories observed at the same time, there is at most one trajectory that corresponds to the sensor data. If the moderate period likelihood for one trajectory is high, the other trajectories should be rejected. This can be well represented by the softmax function [23], which is often used as an activation function in DNNs, so we updated an arbitrary $q_{k}$ using the softmax function as the following equation.

(3) $$q_{k}\to softmax\left(q_{k}\right).$$

We take the interval where the trajectory occurrence time overlaps in $q_{k}$ and perform softmax processing on the corresponding stamp.

#### 3.3.2. Update Rule 2—Sequential Trajectories

During observation, it frequently happens that a person’s trajectory is fragmented for some reason. If there is a trajectory that ends just before another trajectory begins, we can expect that the two trajectories belong to the same person. To reflect this situation, if there is a trajectory that ends immediately before the start of another trajectory, we increase the likelihood of the first trajectory immediately after the start of the second trajectory.

(4) $$q_{k}\to q_{k}+\alpha \left(\Delta t\right)\cdot q_{k-1}.$$

Note that $q_{k-1}$ is the moderate-period likelihood obtained from the trajectory that ended just before $q_{k}$ started, and α(Δt) is a function that decreases with the length of Δt, the time between the previous end and the immediate start. In the example shown in Figure 8, the value $\Delta t=t_{3}-t_{2}^{\prime }$ is applied to $Q_{3}$, which starts immediately after $Q_{2}$ ends.

#### 3.3.3. Integration of Moderate-Period Likelihood *Q* into Full-Period Likelihood *R*

**Figure 9:**
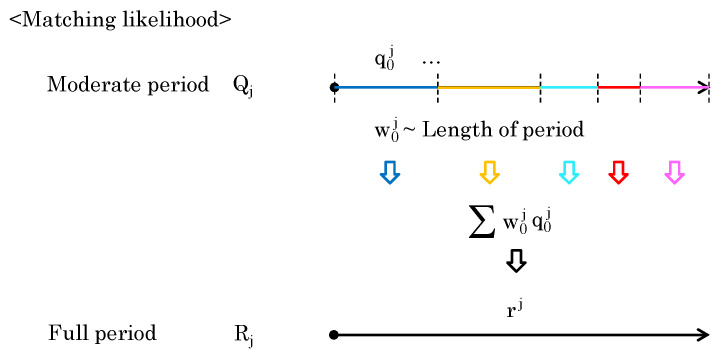
Computation of $R_{j}$ by considering the lengths of the elements of $Q_{j}$.

To compute the full-period likelihood $R_{j}$, consider integrating a set of moderate-period likelihoods $Q_{j}$. Each element of $Q_{j}$ is obtained from different period lengths. When the likelihood of an element of longer period is high, the likelihood of the full period can also be considered high. Conversely, even if the moderate-period likelihood of the element is high, the likelihood may not indicate a trend for the entire dataset. Therefore, the period length should be reflected in the calculation of the full-period likelihood. As shown in Figure 9, the period length $w_{0}^{j}$ of moderate-period likelihood $q_{0}^{j}$ is directly used as a weight to obtain $r^{j}$.

(5) $$r_{j}=\frac{\sum_{q_{i}^{j}\in Q_{j}}w_{i}^{j}\cdot q_{i}^{j}}{\sum w_{i}^{j}}.$$

After the likelihood is obtained for each trajectory $R_{j}$, the trajectory with the largest likelihood is adopted in order according to the winner-takes-all rule. A trajectory that overlaps in time with the set of trajectories that have already been adopted is not adopted. Trajectories with likelihoods below a certain threshold are also not adopted. Trajectories of extremely short periods are excluded from the likelihood calculation at the initial stage and are also not adopted.

Through the combination of our proposed deep learning model and the processing method of the results as described above, we can obtain accurate matching results. In the experimental section, we present detailed experimental results and analysis.

## 4. Experiment

### 4.1. Original Dataset—Long and Short Datasets

#### 4.1.1. Environment and Hyper-Parameters

The search ranges of hyper-parameters in our model are as follows: The learning rate of SyncScore is searched in $7.0\times 10^{-5}$, $1.0\times 10^{-4}$, and $3.0\times 10^{-4}$. The number of SecAttention N is searched in 1, 2, 3, and 4, and the number of heads in multi-attention is searched in 4 and 8. After tuning, we utilize the hyper-parameters with the best performance on the validation set as follows: The learning rate of SyncScore is $1.0\times 10^{-4}$. The number of layers of SecAttention is two and the number of heads is eight. All training uses the AdamW optimizer. The experiment use Binary Cross-Entropy (BCE) as the loss function. The parameters for all the baselines are tuned to attain the best performance or set as proposed by the authors. The experiments used Pytorch on Nvidia GeForce RTX 3090 (24 GB memory).

#### 4.1.2. Long and Short Datasets and Evaluation Algorithm

In this experiment, we confirmed the accuracy of matching trajectories with sensor data. We could not find any existing datasets suitable for our experiments. Therefore, we first created a dataset specifically designed for our purposes and then conducted our experiments on it. (This empirical study was conducted with the approval of the Ethics Committee for Research Involving Humans at the Faculty of Engineering, University of Yamanashi.)

We prepared two datasets: a *Long Dataset* for trajectories and sensor data without video as a training model, and a *Short Dataset* of trajectories and sensor data with perfect tags by manually observing videos for observing the results (Table 1). The proportion of the two labels in the Long Dataset is 56% and 44%.

For the Long Dataset, we manually give the correct answer whether the person with a certain sensor Si matches each full period $R_{j}$ or not, so we can examine the accuracy of the match between $R_{j}$ and $S_{i}$. For the Long Dataset, we compare the scores of the ablation study with and without the proposed Fusion Feature. An ablation study is also performed for SecAttention and the Transformer on which it is based. A case is True Positive (TP) when it is estimated to match $R_{j}$ and $S_{i}$ of the same person, and False Negative (FN) when it is estimated not to match $R_{j}$ and $S_{i}$. A case is False Positive (FP) when a match is estimated for $R_{j}$ and $S_{i}$ of different persons, and True Negative (TN) when no match is estimated. When these are obtained for all combinations of $R_{j}$ and $S_{i}$, Accuracy, Precision, Recall (Sensitivity), and F1 are obtained by the following equations, respectively.

$$\begin{array}{ccc} Accuracy & = & \frac{TP+TN}{TP+TN+FP+FN},\\ Precision & = & \frac{TP}{TP+FP},\\ Recall & = & \frac{TP}{TP+FN},\\ F1 & = & \frac{2\times Recall\times Precision}{Recall+Precision}. \end{array}$$

From the Short Dataset, we can examine whether the proposed Likelihood Fusion Algorithm introduced in Section 3.3 contributes to integrating the trajectories $Q_{j}$ and $R_{j}$ with an appropriate likelihood. The individual effectiveness of Update Rules 1 and 2 in the Likelihood Fusion Algorithm is also examined by ablation studies.

#### 4.1.3. Data Acquisition

To obtain the data, we asked several subjects to wear wearable sensors and to move around the room. For the wearable sensor data, we utilized the WitMotion WT901SDLC sensor, which was worn on each person’s wrist to record movement. The sensor collected nine-axis data such as acceleration, angular velocity, angle, and timestamp. The sampling frequency of the sensor was set to 20 Hz. As for the human trajectory data, we employed a DepthAI camera Luxonis OAK-D-Pro along with the person-detection-retail-0013 model from the OpenVINO library to detect people and obtain their 3D coordinates. The process involved comparing each captured object box with the previous frame, concatenating the obtained points to form a trajectory, and manually annotating the trajectory.

For tagging data, we could have tagged the images but, for this experiment, we first used a simpler tagging method: we assigned a chair to each subject, visualized the trajectory in 3D, and identified the trajectories of each subject sitting in a certain chair and walking around the room before and after sitting in the chair. This method enabled the tagging of trajectories and sensor data for a long time period without the cumbersome task of video tagging. For the *Long Dataset*, we collected this data over 2.5 h and we employed a five-fold cross-validation approach for training the model.

To construct a more precise *Short Dataset*, we utilized camera videos to collect precisely corresponding data. When the videos could be viewed together, we could tag which person had the wearable sensor. Even after the trajectories collided, we could estimate which trajectory was made by the target person. With this method, it is necessary to match the images and trajectories at each period. Although it is possible to create a definite correct ground truth, the task of tagging becomes difficult. We used this dataset only for the test and did not use it for training and validation. The captured images were set to a resolution of 544 × 320 and sampled at a frequency of 7 Hz. There were ten videos in total, with two cameras simultaneously capturing five different situations from different positions. Four people in the video were wearing wearable sensors. So, we can say that there are 40 samples to observe the results. The average length of the videos was 52 s.

In our study, we classified the results in the same way as a typical classification task and validated them using metrics such as F1 score, accuracy, precision, and recall. Table 2 shows the performance metrics of the SyncScore network, which calculates the matching likelihood per unit period. Here, the process is performed at the unit period level *P*, before integrating to moderate period *Q* or full period *R*. Recognition is considered successful when the correct trajectory shows the highest likelihood compared to other trajectories at the same period. Since there are no models similar to our task for comparison, we compared the results when each method was removed by an ablation study.

#### 4.1.4. Long Dataset

In Figure 4 of Section 3.2, a Fusion Feature is created by concatenating the trajectory and sensor data. Even without creating this vector, the input and output of the network can be the same. In the experiment, we obtained the score when this vector was omitted. Also, since we could use Transformer instead of SecAttention, we also obtained the score in this case. As a result, it was confirmed that the score was best when both elements were included. From this, it can be said that both Fusion Feature and SecAttention are effective for recognizing our dataset.

**Figure 10:**
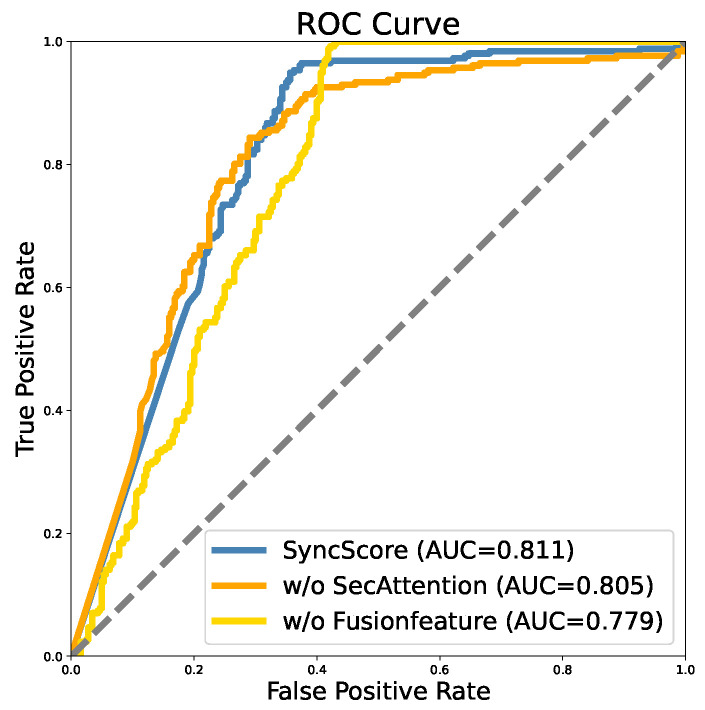
Ablation results of ROC curve in *Long Dataset*.

In Figure 10, the AUC value of the SyncScore model stands at 0.811, surpassing the values of 0.805 observed when excluding the SecAttention module and 0.779 when omitting the Fusion Feature module. This comparison underscores the superior performance of the SyncScore model in accurately classifying positive samples and distinguishing them from negative ones. The model excels not only in correctly identifying truly positive samples but also in minimizing misclassifications of negative samples as positive. The ROC curve of the SyncScore model is closer to the upper left corner, indicating that it performs better across the entire FPR range. This means that the SyncScore model can achieve a higher TPR at a lower FPR, thereby improving the overall performance of the model. The slope of the ROC curve is steep, which also shows that the recall metric performs well. Based on these, it is evident that the Fusion Feature and SecAttention exert a discernible impact on the overall performance of the model.

In our study aimed at advancing the field of trajectory matching, we have strategically chosen to compare the Transformer model [17], along with its advancements in the form of Informer [24] and GTN (Gated Transformer Network) [25], to better understand their applicability and performance in this context. The decision to compare the Informer and GTN models to our work stems from their inherent capabilities in handling sequence and time-series data, which are fundamentally related to trajectory analysis. This involves the utilization of our novel deep learning model, SecAttention, an improvement based on the Transformer, tailored for trajectory matching.

The Transformer model, with its self-attention mechanism, serves as an excellent baseline for analyzing sequence data, prompting its inclusion in our comparative analysis. The Informer builds upon the Transformer by adding convolutional layers to better handle sequence dependencies, crucial for precise trajectory matching. Meanwhile, GTN brings a specialized approach to multivariate time series by blending a gating mechanism with self-attention, adeptly extracting key information for trajectory matching.

Given that our SecAttention model is an extension of the Transformer, focusing our comparative analysis on this component offers a direct evaluation of its enhancements. By replacing the SecAttention part of our SyncScore model with each comparison method in turn, we aim to assess their relative effectiveness strictly within the context of our model’s framework.

As detailed in Table 3, although our method slightly trails behind GTN in terms of precision, it excels in all other metrics, notably achieving the highest F1 score. This outcome underscores the efficacy of our approach in trajectory matching, highlighting the significance of our improvements over the foundational Transformer model.

#### 4.1.5. Short Dataset

Table 4 shows the results for the full period R. For all combinations of trajectories and sensor data in the dataset, the proposed method showed an accuracy of 0.725. Note that the scores for accuracy, precision, recall, and F1 are all the same because, when one trajectory is adopted, another is rejected, resulting in the same false negative and false positive values in most cases. When Update Rules 1 and 2, shown in Section 3.3, were not applied, this accuracy decreased. Although the accuracy of the proposed method cannot be deemed sufficiently high, we were able to demonstrate the effectiveness of each of Update Rules 1 and 2.

Table 5 shows the comparison results of applying different models in the *Short Dataset*. Transitioning from Transformer to Informer, and subsequently to GTN, fluctuations in accuracy were observed. Here, we adopted the update method of Rules 1 and 2. However, upon introducing SecAttention, a significant improvement in performance was noted. More importantly, after integrating SecAttention into the SyncScore model, the accuracy reached the highest point, indicating that the addition of SecAttention can significantly enhance the model’s ability to process time-series data. This result proves the effectiveness of SyncScore in improving the accuracy of trajectory-matching, especially in application scenarios with detailed temporal alignment.

**Figure 11:**
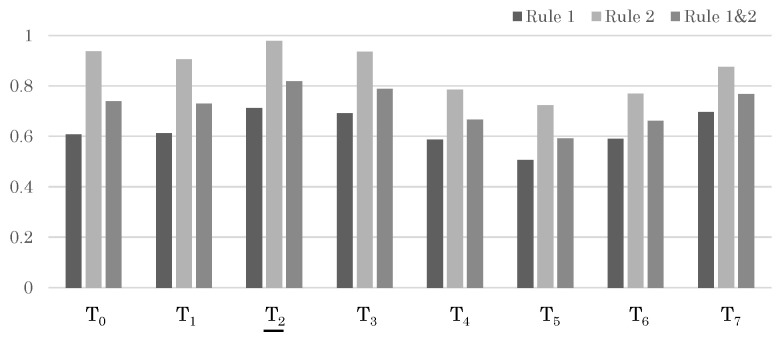
Likelihood results for each trajectory in Sample 1. The trajectories that should be matched correctly are indicated with underlines.

**Figure 12:**
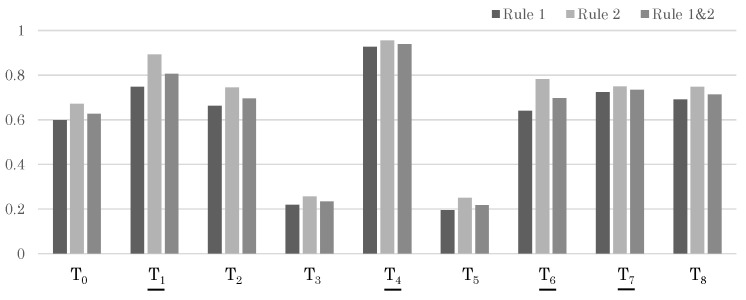
Likelihood results for each trajectory in Sample 2. The trajectories that should be matched correctly are indicated with underlines.

**Figure 13:**
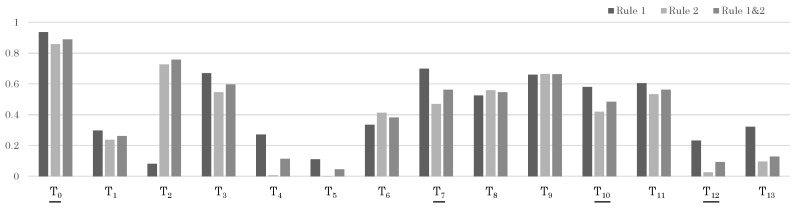
Likelihood results for each trajectory in Sample 3. The trajectories that should be matched correctly are indicated with underlines.

Table 6, Table 7 and Table 8 and Figure 11, Figure 12 and Figure 13 show detailed results for the three test samples. These test samples do not have corresponding videos, and the correct trajectories are given only by clues to the seats. In each case, five subjects were observed, and a set of correct trajectories was defined.

For Test Sample 1, each person produced a set of trajectories, and the sensor data came from the wearable sensor of the target person in Trajectory Set 3. Therefore, if the likelihood of Trajectory Set 3 = $\left\{T_{2}\right\}$ was higher than that of other trajectory sets, it was considered a successful recognition. First, the likelihood at the moderate period *Q* is shown in Figure 11. At this point, even without applying the update rules, the correct trajectory $T_{2}$ shows the maximum likelihood. Furthermore, the likelihood for the full period *R* is shown in Figure 6. Here, again, the likelihood for the correct trajectory set, Trajectory Set 3, is the highest, indicating a successful recognition.

Table 7 and Figure 12 show similar results for a different test sample, Test Sample 2. In this sample, the correct Trajectory Set 2 is divided into $T_{1},T_{4},T_{6},$ and $T_{7}$. Although each shows a high likelihood individually, there are also individual trajectories with even higher likelihoods. However, when viewed as a set of trajectories, the correct Trajectory Set 2 shows the highest likelihood, and we are finally able to obtain the correct result.

From Table 8 and Figure 13, it can be observed that the correct target set is Set 1. Although certain individual trajectories, such as $T_{8}$, $T_{9}$, and $T_{11}$, may exhibit high likelihoods when considered in isolation, when we view these trajectories as a collective (namely Set 2), we find that, as a whole, this set demonstrates low likelihood. This analytical approach, considering combinations of trajectories rather than individual ones, can ultimately assist us in deriving the correct conclusions regarding Sample 3. We also note that the sole application of Rule 1 has led to misjudgment of the target, which is attributable to the incomplete considerations in the calculation under Rule 1. This inadvertently underscores the efficacy and importance of applying both Rule 1 and Rule 2 concurrently.

**Figure 14:**
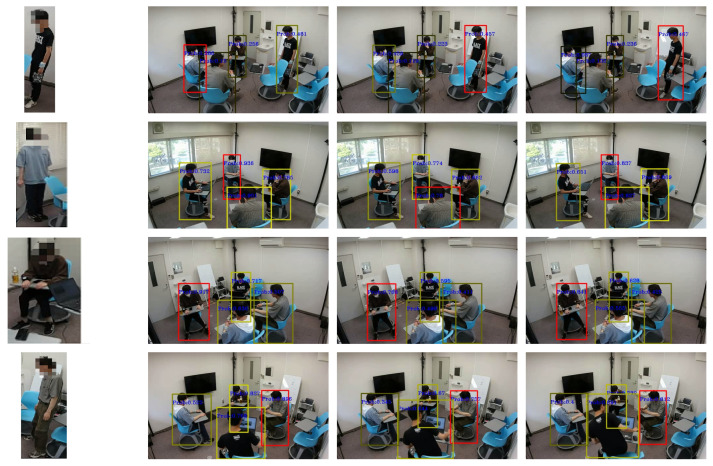
Visualization of integration results. Each row is a different video visualization result: (**left**) target, (**right-first**) the result of Update Rule 2 processing, (**right-middle**) the result of Update Rule 1 processing, and (**right-most**) the result of the fusion of both update rules.

In Figure 14, we show a visualization of part of the experiment. The red box indicates the matching target of the data, and the color depth of the other boxes indicates the probability of the matching result. The darker the color, the lower the matching value, and vice versa. In each of the examples shown in the figure, the person wearing the wearable sensor was correctly estimated with Update Rules 1 and 2. Using one method alone produced one false result. The use of both update rules together comprehensively provided more accurate results. For the other people, different likelihoods were obtained depending on the movement of the trajectory. The correct person was extracted regardless of the position in the image or the depth position.

In some samples, we observe that results obtained from alternative trajectory sets closely resemble the correct ones. This occurrence is due to the similarity in behavioral patterns among multiple targets, resulting in a convergence of their likelihoods owing to analogous behavioral models.

From the above, it can be concluded that, for the aforementioned samples, the likelihood for individual trajectories is being calculated somewhat correctly and, furthermore, the integration of likelihoods works well.

### 4.2. UEA Dataset

The UEA Time Series Classification Dataset [26] is a publicly available dataset designed for time-series classification research, developed and maintained by the University of East Anglia (UEA). It consists of several time-series datasets covering various domains, including health monitoring (Atrial Fibrillation), motion recognition (Basic Motions), speech recognition (JapaneseVowels), and environmental sensing (ERing), among others. These datasets allow researchers to test and compare algorithm performance in different application scenarios.

The time series in the UEA dataset have different lengths, sampling frequencies, and data types, which provide diverse challenges and requirements for data processing. Although there are no pairs of data from wearable sensors and human trajectories in videos such as those addressed in this work, this is a dataset that has collected many synchronized pairs of time-sequential signals from different modalities. Each dataset is accompanied by well-defined class labels that facilitate the evaluation of classification tasks. This standardized labeling allows for direct performance comparison among different algorithms. To evaluate the recognition and integration algorithms proposed in this study, we performed experiments on this dataset and compared them with conventional methods.

In our experiments, we used the UEA dataset for comparison and the accuracy rate was used as an indicator for comparison. Since our model is a dual-input model, which is different from the data in the dataset, we use the same data as input for experiments. The methods involved included ShapeNet [27] for time-series classification, which uses a method combining neural network and shapelet; TapNet [28], which represents metric learning methods and small-sample learning; DTW [29], which represents distance-based methods, 1NNI, which stands for Independent Wrapping, where each dimension is calculated separately and then summed; and 1NND, which stands for Dependent Wrapping, for all Dimensions for DTW calculations. We also used clustering methods for classification, such as K-Means; and DSTP [30], GeoMAN [31], and STAM [32] for spatiotemporal data modeling and forecasting tasks. From the results in Table 9, it is observable that our method achieves the best win/tie results among all methods.

In our experiments, we compared our proposed module with traditional time-series classification methods based on accuracy metrics. The results demonstrate a performance improvement of our module on the UEA Time Series Classification Dataset. Our module effectively captures the temporal correlations and feature representations in the time-series data compared to previous methods, resulting in enhanced classification accuracy and performance. These experimental findings further validate the effectiveness and generality of our proposed module, which is applicable to our specific task and to common single-input multidimensional time-series processing tasks. It presents a new approach and perspective for time-series data analysis and classification research.

## 5. Conclusions

In this paper, we propose a method for identifying human trajectories obtained from cameras and other devices by matching the trajectories to peoples’ wearable sensor data. People who were given sensor data from wearable devices were shown which trajectories corresponded to their own trajectories and could use that data to analyze their behavior independently. By checking the match between sensor data and trajectories, without using camera images as clues, a desirable system can be derived.

The problem with this method is that human trajectory data and sensor data have different modalities, and incomplete trajectory data are often obtained, making conventional matching methods inapplicable. To solve these problems, we proposed the SyncScore model to obtain the matching likelihood for short-term period data. We then used the Likelihood Fusion algorithm to gradually update the likelihood. We confirmed that the proposed methods gave high scores in our original dataset. We also confirmed that the proposed method provides better accuracy on the UEA dataset than the conventional method.

Our future work will include investigating how well the trajectories can be identified in large spaces with several people. One of the characteristics of AI cameras is their capability to obtain 3D trajectories by focusing the range to a certain distance. For this reason, it is possible that, in a large space, a person might be captured by a camera without their trajectory being found. Also, the presence of a large number of people will often occlude them from each other. In such cases, the trajectory would not score as highly as it would have in this work. An additional algorithm is required to deal with such incomplete trajectories. We believe that, even if the accuracy is not perfect, it can still be used. Since the results are given together with the likelihood, it is possible not to present the results to the user if the likelihood is not sufficient.

The time synchronization between the wearable sensor and the AI camera was assumed to be perfect in our experiment, but this was difficult to confirm. It is generally assumed that the AI camera is always connected to the network, but the time synchronization of the wearable sensor may be imperfect. It may be possible to synchronize the data by detecting the movements of the wearable sensors and detecting the targets’ trajectories before comparing the data. We would like to add a time synchronization algorithm using only data. This is a challenging problem in itself, because it requires solving the problem without knowing which person’s sensor data is being observed.

For completely unknown data that is not included in the current dataset, it is possible that sufficient accuracy may not be obtained if the camera capturing conditions or the behavior of the person in the video are very different.

The Nvidia GeForce RTX 3090 (24 GB) used in the experiments is sufficiently high-spec, but the processing time is about twice the length of the video, so it does not work in real time. The system does not acquire wearable sensor signals via the network currently, and we have to directly connect the cable to retrieve the signals after the experiment is over, which is also an issue in operation.

## Figures and Tables

**Table 1:** Information on two datasets: a *Long Dataset* without video, and a *Short Dataset* with video and manually added tags.

*Long Dataset*	Train Size	Test Size	Number of Dimensions
Trajectory data	5780	990	3
Sensor data	5780	990	9
***Short Dataset***	**People with Sensor**	**Video Length**	**Number of Videos**
Information	4	52 s	10

**Table 2:** Evaluation results in *Long Dataset*.

Fusion Feature		✔	✔	✔
Transformer			✔	
SecAttention	✔			✔
Accuracy	0.708	0.737	0.700	**0.763**
Precision	0.642	0.663	0.639	**0.670**
Recall	0.773	0.880	0.918	**0.921**
F1	0.702	0.756	0.752	**0.776**

**Table 3:** Ablation results on trajectories in *Long Dataset*.

Method	Transformer	Informer	GTN	Ours
Accuracy	0.700	0.742	0.759	**0.763**
Precision	0.639	0.647	**0.674**	0.670
Recall	0.918	0.912	0.876	**0.921**
F1	0.752	0.757	0.762	**0.776**

**Table 4:** Evaluation results on videos in *Short Dataset*.

Update Rule 1	✔		✔
Update Rule 2		✔	✔
Accuracy	0.595	0.575	**0.725**
Precision	0.595	0.575	**0.725**
Recall	0.595	0.575	**0.725**
F1	0.595	0.575	**0.725**

**Table 5:** Matching results of videos in *Short Dataset*.

Method	Transformer	Informer	GTN	Ours
Accuracy	0.700	0.675	0.700	**0.725**
Precision	0.700	0.675	0.700	**0.725**
Recall	0.700	0.675	0.700	**0.725**
F1	0.700	0.675	0.700	**0.725**

**Table 6:** Likelihood results of trajectory set for Sample 1. Bold set is the correct result.

Trajectory Set	Rule 1	Rule 2	Rules 1 and 2
Set 1: $T_{0}$	0.6076	0.9375	0.7395
Set 2: $T_{1}$	0.6127	0.9059	0.7300
**Set 3: $T_{2}$**	**0.7121**	**0.9790**	**0.8189**
Set 4: $T_{3}$	0.6921	0.9344	0.7890
Set 5: $T_{4},T_{5},T_{6},T_{7}$	0.5949	0.7877	0.6720

**Table 7:** Likelihood results of trajectory set for Sample 2.

Trajectory Set	Rule 1	Rule 2	Rules 1 and 2
Set 1: $T_{0}$	0.5981	0.6718	0.6276
**Set 2:** $T_{1},T_{4},T_{6},T_{7}$	**0.7595**	**0.8454**	**0.7939**
Set 3: $T_{2},T_{8}$	0.6770	0.7467	0.7048
Set 4: $T_{3}$	0.2193	0.2559	0.2340
Set 5: $T_{5}$	0.1954	0.2500	0.2172

**Table 8:** Likelihood results of trajectory set for Sample 3.

Trajectory Set	Rule 1	Rule 2	Rules 1 and 2
**Set 1**: $T_{0},T_{7},T_{10},T_{12}$	0.6693	**0.8083**	**0.7249**
Set 2: $T_{1},T_{8},T_{9},T_{11}$	0.3621	0.4019	0.3780
Set 3: $T_{2},T_{13}$	**0.6815**	0.7715	0.7175
Set 4: $T_{3},T_{4},T_{5},T_{6}$	0.3641	0.3601	0.3625

**Table 9:** Accuracy for UEA.

	ShapeNet	TapNet	DTW-1NND	DTW-1NNI	DSTP-p	DSTP	GeoMAN	GeoMAN-g	GeOMAN-l	STAM	Ours
ArticularyWordRecognition	0.987	0.987	0.987	0.980	0.846	0.850	0.920	0.906	0.923	0.970	**0.990**
AtrialFibrillation	0.400	0.333	0.220	0.200	0.400	**0.600**	0.400	0.467	0.333	0.533	0.467
BasicMotions	**1.000**	**1.000**	0.975	**1.000**	0.800	0.875	0.950	0.950	0.925	0.675	**1.000**
CharacterTrajectories	0.980	**0.997**	0.989	0.969	0.060	0.060	0.060	0.060	0.060	0.060	0.994
Cricket	0.986	0.958	**1.000**	0.986	0.153	0.194	0.194	0.208	0.194	0.750	0.992
DuckDuckGeese	**0.725**	0.575	0.600	0.550	0.280	0.260	0.360	0.400	0.380	0.420	**0.725**
EigenWorms	**0.878**	0.489	0.618	-	0.420	0.420	0.420	0.420	0.420	0.412	0.592
Epilepsy	0.987	0.971	0.964	0.978	0.384	0.384	0.333	0.341	0.326	0.565	**0.989**
EthanolConcentration	0.312	0.323	0.323	0.304	0.297	0.357	0.327	0.312	0.323	0.308	**0.512**
ERing	0.133	0.133	0.133	0.133	0.478	0.441	0.426	0.463	0.459	0.692	**0.865**
FaceDetection	0.602	0.556	0.529	0.500	0.518	0.515	0.517	0.517	0.630	**0.650**	0.528
FiMovements	0.580	0.530	0.530	0.520	0.530	**0.620**	0.620	0.530	0.520	0.560	0.585
HMDirection	0.338	0.378	0.231	0.306	0.487	0.378	**0.527**	0.473	0.392	**0.527**	0.480
Handwriting	**0.451**	0.357	0.286	0.316	0.055	0.051	0.051	0.037	0.051	0.099	0.185
Heartbeat	0.756	0.751	0.717	0.658	0.722	0.722	0.722	0.722	0.727	0.756	**0.772**
InsectWingbeat	0.25	0.208	-	-	0.010	0.010	0.010	0.010	0.010	0.010	**0.614**
JapaneseVowels	**0.984**	0.965	0.949	0.959	0.084	0.084	0.084	0.084	0.084	0.084	0.971
Libras	0.856	0.850	0.870	**0.894**	0.201	0.228	0.233	0.272	0.172	0.589	0.500
LSST	**0.590**	0.568	0.551	0.575	0.315	0.315	0.315	0.315	0.315	0.316	0.510
MotorImagery	0.610	0.590	0.500	-	0.560	0.520	**0.630**	0.590	0.560	0.560	0.560
NATOPS	0.883	**0.939**	0.883	0.850	0.233	0.228	0.250	0.333	0.522	0.767	0.910
PEMS-SF	**0.751**	**0.751**	0.711	0.734	0.168	0.145	0.162	0.671	0.688	0.746	0.746
PenDigits	0.977	**0.980**	0.977	0.939	0.112	0.110	0.331	0.350	0.384	0.888	0.837
Phoneme	**0.298**	0.175	0.151	0.151	0.037	0.059	0.050	0.068	0.042	0.060	0.137
RacketSports	0.882	0.868	0.803	0.842	0.283	0.283	0.290	0.290	0.336	0.441	**0.892**
SelfRegulationSCP1	0.782	0.652	0.775	0.765	0.580	0.604	0.580	0.563	0.870	**0.877**	0.800
SelfRegulationSCP2	0.578	0.550	0.539	0.533	0.561	0.539	0.567	0.544	0.561	0.556	**0.580**
SpokenArabicDigits	0.975	**0.983**	0.963	0.959	0.010	0.010	0.010	0.010	0.010	0.010	0.940
StandWalkJump	**0.533**	0.400	0.200	0.333	0.467	0.333	0.467	0.467	**0.533**	**0.533**	**0.533**
UWaveGestureLibrary	**0.906**	0.894	0.903	0.868	0.406	0.497	0.466	0.375	0.444	0.813	0.813
*Wins/Ties*	10	6	1	2	0	2	2	0	1	4	**11**
*Average Rank*	**2.7**	3.8	5.3	6.3	7.4	7.4	6.4	6.8	6.5	5.0	2.9

## Data Availability

The raw data supporting the conclusions of this article will be made available by the authors on request.
